# Interaction between HLA-DRB1-DQB1 Haplotypes in Sardinian Multiple Sclerosis Population

**DOI:** 10.1371/journal.pone.0059790

**Published:** 2013-04-08

**Authors:** Eleonora Cocco, Raffaele Murru, Gianna Costa, Amit Kumar, Enrico Pieroni, Cristina Melis, Luigi Barberini, Claudia Sardu, Lorena Lorefice, Giuseppe Fenu, Jessica Frau, Giancarlo Coghe, Nicola Carboni, Maria Giovanna Marrosu

**Affiliations:** 1 Multiple Sclerosis Center, Department of Public Health and Clinical and Molecular Medicine, University of Cagliari, Cagliari, Italy; 2 CRS4 Science and Technology Park Polaris - Piscina Manna, Pula (CA), Italy; Charite Universitätsmedizin Berlin, Germany

## Abstract

We performed a case-control study in 2,555 multiple sclerosis (MS) Sardinian patients and 1,365 healthy ethnically matched controls, analyzing the interactions between HLA-DRB1-DQB1 haplotypes and defining a rank of genotypes conferring a variable degree of risk to the disease. Four haplotypes were found to confer susceptibility (*13∶03-*03∶01 OR = 3.3, Pc 5.1×10^−5^, *04∶05-*03∶01 OR = 2.1, Pc 9.7×10^−8^, *15∶01-*06∶02 OR = 2.0, Pc = 9.1×10^−3^, *03∶01-*02∶01 OR = 1.7 Pc = 7.9×10^−22^) and protection (*11, OR = 0.8, Pc = 2.7×10^−2^, *16∶01-*05∶02 OR = 0.6, Pc = 4.8×10^−16^, *14∶01-4-*05∶031 = OR = 0.5, Pc = 9.8×10^−4^ and *15∶02-*06∶01 OR = 0.4, Pc = 5.1×10^−4^). The relative predispositional effect method confirms all the positively associated haplotypes and showed that also *08 and *04 haplotypes confers susceptibility, while the *11 was excluded as protective haplotype. Genotypic ORs highlighted two typologies of interaction between haplotypes: i) a neutral interaction, in which the global risk is coherent with the sum of the single haplotype risks; ii) a negative interaction, in which the genotypic OR observed is lower than the sum of the OR of the two haplotypes. The phylogenic tree of the MS-associated DRB1 alleles found in Sardinian patients revealed a cluster represented by *14∶01, *04∶05, *13∶03, *08∶01 and *03∶01 alleles. Sequence alignment analysis showed that amino acids near pocket P4 and pocket P9 differentiated protective from predisposing alleles under investigation. Furthermore, molecular dynamics simulation performed on alleles revealed that position 70 is crucial in binding of MBP 85–99 peptide. All together, these data suggest that propensity to MS observed in Sardinian population carried by the various HLA-DRB1-DQB1 molecules can be due to functional peculiarity in the antigen presentation mechanisms.

## Introduction

Multiple sclerosis (MS) is a common neurological inflammatory and degenerative disease of young adulthood, whose predisposition is widely attributed to an interplay of genetic and environmental factors [1]–[4]. The genetic component of the disease is conferred by a rather large number of small genetic variants, as recently identified by a genome wide association study [4], with the main genetic determinant located at the human leukocyte antigen (HLA) class II DRB1 and DQB1 loci. Despite the fact that the HLA-DRB1*15 haplotype (DRB1*15∶01-DQA1*01∶02-DQB1*06∶02) represents the main disease risk factor in populations of North European origin [4], several different allelic associations have been identified in South European populations [5]–[7], in Israel [8], and other secondary DRB1 allelic associations have been found in North European populations [4]. In MS populations of North European ancestry, several studies have determined the presence of alleles conferring resistance and influencing predisposition to the disease [9]–[12]. For instance, the effect of the *15∶01 allele, which maximally increases the MS risk in white populations of Northern-European descent [4], is either cancelled by the co-presence of the *14 allele, or is reinforced by the co-presence of the *08 allele [10]–[12]. Sardinia is a major Italian island with a high incidence of MS [13], [14], distinguished by a unique, highly homogeneous genetic make-up, resulting from fixation of alleles and haplotypes that are rare or absent elsewhere [15]. A significant positive association with MS and five DRB1-DQB1 HLA haplotypes, including the *13∶03-*03∶01, *04∶05-*03∶01, *03∶01-*02∶01, *04∶05-*03∶02 and *15∶01-*06∶02 have been reported in the Sardinian population, with different ranges of risk carried by patients/individuals with each associated haplotype [16]. The independence of associated haplotypes was recently assessed together with the presence of negatively associated haplotypes [17]. However, interactions between the negatively and positively associated haplotypes were not assessed in Sardinian MS patients [17]. As reported in other populations [9]–[12], interactions between alleles or haplotypes modulate risk of the disease due to HLA class II variants, thus determining the global risk carried by the individual genotype. Moreover, such interactions would help to gain some insight in molecular mechanisms at the basis of the immune response modulation by specific HLA alleles.

In the present study we have analyzed the HLA class II haplotypic and genotypic risk in Sardinian MS patients, with the specific aim to define whether trans-interactions between DRB1-DQB1 haplotypes concur in modifying the risk of the disease. For this, we have first defined the haplotypic risk using a large case-control association analysis, evaluating the odds ratio (OR) values for each haplotype. As several DRB1-DQB1 variants were positively and negatively associated with the disease, cases and controls were analyzed to establish the predisposition, protective, or neutral effects of DRB1-DQB1 haplotype using the relative predispositional effect (RPE) method [18]. Indeed, when one or more alleles showed a strong association with a given disease, as in the case of Sardinian MS population, it was difficult to assess whether a decrease of one allele (or more) is a true negative association or an expected consequence of the increased frequency of a different (or more) alleles. Thereafter, the effect of interactions between haplotypes was analyzed.

Genotypic OR values showed two kinds of interactions: neutral and negative, which was then described through an empirical mathematical model. Finally, sequence alignment and structural-dynamical analysis of the predisposing and protective DRB1 haplotype were performed, which showed two main allele clusters, the already described DR2 group [17] and a second one represented by the protective *14∶01 and the predisposing *03∶01, *04∶05, *08∶01 and *13∶03 alleles. Molecular modeling studies carried out in the latter group, suggested position 70 which is located at the P4 pocket play a significant role in antigen binding that could be functionally linked to disease protection or predisposition.

## Results

### Analysis of Associated HLA-DRB1-DQB1 Haplotypes

The association of DRB1-DQB1 haplotypes and genotypes was examined in 2,555 Sardinian MS patients (for a total chromosome number of 5,110) and 1,365 controls (total chromosome number of 2,730). Only haplotypes represented in at least 1% of the sample were considered. After performing correction for the 15 considered haplotypes, four of them were found to be significantly positively associated: *13∶03-*03∶01 (OR = 3.3, 95% CI 1.9–5.6, Pc = 5.1×10^−5^); *04∶05- *03∶01 (OR = 2.1, 95% CI 1.6–2.6, Pc = 9.7×10^−8^); *15∶01-*06∶02 (OR = 2.0, 95% CI 1.3–3.0 Pc = 9.1×10^−3^) and *03∶01-*02∶01 (OR = 1.7, 95% CI 1.5–1.9, Pc = 7.9×10^−22^). A similar analysis showed concurrently that four other haplotypes resulted to be negatively associated: *11 (OR = 0.8, 95% CI 0.7–0.9, Pc 2.7×10^−2^), *16∶01-*05∶02 (OR = 0.6, 95% CI 0.5–0.7, Pc = 4.8×10^−16^), *14∶01-4*05∶031 (OR = 0.5, 95% CI 0.4–0.7, Pc = 9.8×10^−4^) and *15∶02-*06∶01 (OR = 0.4, 95% CI 0.3–0.7, Pc = 5.1×10^−4^). Data are reported in Table 1.

**Table 1:** Case-control analysis of HLA-DRB1-DQB1 haplotypes from 2,555 multiple sclerosis patients and 1,365 healthy ethnically matched controls. Only haplotypes represented in at least 1% of the sample were considered.

Haplotypes	MS Patients	*%*	Controls	*%*	OR	95% C.I.		P*c*
*13∶03–*03∶01	97	1.9	16	0.6	3.3	1.9	5.6	5.1×10^−05^
*04∶05–*03∶01	306	6.0	82	3.0	2.1	1.6	2.6	9.7×10^−08^
*15∶01–*06∶02	114	2.2	31	1.1	2.0	1.3	3.0	9.1×10^−03^
*08	71	1.4	21	0.8	1.8	1.1	3.0	2.3×10^−01^
*03∶01–*02∶01	1680	32.9	607	22.2	1.7	1.5	1.9	7.9×10^−22^
*04	576	11.3	312	11.4	1.0	0.9	1.1	
*13	114	2.2	69	2.5	0.9	0.7	1.2	
*11	656	12.8	420	15.4	0.8	0.7	0.9	2.7×10^−02^
*01	366	7.2	238	8.7	0.8	0.7	1.0	2.1×10^−01^
*07	197	3.9	143	5.2	0.7	0.6	0.9	6.3×10^−02^
*12∶01–*03∶01	56	1.1	44	1.6	0.7	0.5	1.0	7.9×10^−01^
*10∶01–*05∶01	80	1.6	68	2.5	0.6	0.4	0.9	6.2×10^−02^
*16∶01–*05∶02	603	11.8	513	18.8	0.6	0.5	0.7	4.8×10^−16^
*14∶01-4–*05∶031	77	1.5	77	2.8	0.5	0.4	0.7	9.8×10^−04^
*15∶02–*06∶01	45	0.9	54	2.0	0.4	0.3	0.7	5.1×10^−04^
	5038		2695					
Total	5110		2730					

Pc = P corrected for the 15 considered haplotypes. NS = not significant.

Rare haplotypes belonging to the same haplogroup were grouped together: as *11 were designed *11∶01-02-03-04 -*03∶01, *11∶01-*03∶03-*05∶02 and *11∶04-*06∶03; as *07 were designed *07∶01- *02∶01 and *07∶01-*03∶03; as *13 were designed *13∶01-*06∶03-*03∶03, *13∶02-*05∶01-*05∶031-*06∶02-*06∶04-*06∶05-*06∶09, *13∶05-*03∶01 and *13∶16-*06∶04; as *04 were designed *04∶01-*03∶01-*03∶02, *04∶02-*03∶02, *04∶03– *03∶01-02-04-05, *04∶04-*03∶02-*04∶02, *04∶05-*02∶01, *04∶05–*03∶02, *04∶06-*03∶02, *04∶07-*03∶01 and *04∶08-*03∶01; as *08 were designed *08∶01-*04∶02, *08∶03-*03∶01 and *08∶04-*03∶01-*04∶02; as *01 were designed *01∶01 *05∶01, *01∶02-*05∶01 and *01∶03- *05∶01.

The positively and negatively associated haplotypes could be due to a displacement effect; the predisposition, protective, or neutral effects of DRB1-DQB1 haplotype, which was then further established using the RPE method, considering haplotypes of the same data set. Data are showed in Table 2.

**Table 2:** Relative predispositional effect: the overall frequency distribution of all haplotypes at the DRB1-DQB1 loci in MS patients (n = 2,555) compared with the distribution in controls (N = 1,365).

Haplotypes	Observed	Expected	chi2	p - test z	test z	Round
*03∶01–*02∶01	1680	1136	260.29	6.4×10^−25^	10.31	Round 1
*04∶05–*03∶01	306	132	227.26	3.2×10^−15^	7.88	Round 2
*13∶03–*03∶01	97	24	214.69	2.4×10^−10^	6.33	Round 3
*15∶01–*06∶02	114	46	98.79	3.9×10^−07^	5.07	Round 4
*08	71	31	52.995	1.6×10^−04^	3.78	Round 5
*04	576	449	35.65	6.5×10^−05^	3.99	Round 6
*16∶01–*05∶02	603	700	13.40	3.5×10^−03^	2.92	Round 7
*15∶02–*06∶01	45	78	14.11	7.0×10^−03^	2.70	Round 8
*14∶01-4–*05∶031	77	114	11.94	1.5×10^−02^	2.43	Round 9

DRB1-DQB1 haplotypes in MS patients (col.1), the number observed (col. 2) and expected from controls on the basis of the assumption that there were no differential predispositional effects on the DRB1-DQB1 haplotypes (col. 3), and the contribution of each haplotype to the overall **χ**2 (col.4). The overall **χ**2 distribution was considered statistically significant at P<0.001 (col. 5).

Rare haplotypes belonging to the same haplogroup were grouped together: as *04 were designed *04∶01-*03∶01-*03∶02, *04∶02-*03∶02, *04∶03– *03∶01-02-04-05, *04∶04-*03∶02-*04∶02, *04∶05-*02∶01, *04∶05-*03∶02, *04∶06-*03∶02, *04∶07-*03∶01 and *04∶08-*03∶01; as *08 were designed *08∶01-*03∶01-*04∶02, *08∶03-*03∶01 and *08∶04-*03∶01-*04∶02.

The haplotype with the largest contribution to MS susceptibility was found to be the *03∶01-*02∶01 (P = 6.4×10^−25^). After this haplotype was removed, we observed the *04∶05-*03∶01 haplotype to be still significant (P = 3.2×10^−15^), followed by the *13∶03-*03∶01 (P = 2.4×10^−10^), the *15∶01-*06∶02 (P = 3.9×10^−7^), the *08 (P = 1.6×10^−4^) and the *04 (P = 6.5×10^−5^) haplotypes. Once we removed the above mentioned haplotypes, a decreased frequency of the *16∶01-*05∶02 (P = 3.5×10^−3^), *15∶02-*06∶01 (P = 7.0×10^−3^) and *14∶01-4-*05∶031 (P = 1.5×10^−2^) was observed. In particular the *11 haplotype was not confirmed to be negatively associated. The contribution of haplotypes was also examined by multivariate analysis. Some differences in association were found: the positive association with the *08 and with the *04 haplotype found using RPE method was not confirmed using multivariate analysis, which instead showed an association with the *10∶01-*05∶01, *01, *07 and *12∶01-*03∶01 haplotypes. All the other associated haplotypes found using RPE method were confirmed by the multivariate analysis. Data are reported in Table S1.

### Analysis of Associated HLA-DRB1-DQB1 Genotypes

To understand the risk associated with the genotype, we examined genotypic ORs in a case-control analysis, using the same population as in haplotypic case-control study. After correction for the 28 considered genotypes, four of them were found to be significantly positively associated: *03∶01-*02∶01/*13∶03-*05∶01 (OR = 4.3, 95% CI 1.7–11.0, Pc = 2.2×10^−2^), *03∶01-*02∶01/*15∶01-*06∶02 (OR = 3.9, 95% CI 1.8–8.6, Pc = 9.0×10^−3^), *03∶01-*02∶01/*03∶01-*02∶01 (OR = 3.1, 95% CI 2.3–4.0, Pc = 2.0×10^−15^) and *04∶05-*03∶01/*03∶01-*02∶01 (OR = 2.8, 95% CI 1.7–4.5, Pc = 8.1×10^−4^), and five genotypes were found to be negatively associated: *03∶01-*02∶01/*16∶01-*05∶02 (OR = 0.6, 95% CI 0.5–0.8, Pc = 1.6×10^−2^), *16∶01-*05∶02/*11 (OR = 0.6, 95% CI 0.4–0.7, Pc = 3.3×10^−3^ ), *07/*11 (OR = 0.4, 95% CI 0.2–0.7, Pc = 4.4×10^−2^), *16∶01-*05∶02/*16∶01-*05∶02 (OR = 0.3, 95% CI 0.2–0.4, P = 6.0×10^−7^) and *14∶01-4-*05∶031/*16∶01-*05∶02 (OR = 0.2, 95% CI 0.1–0.5, Pc 1.1×10^−3^). The supporting data are reported in Table 3.

**Table 3:** Case-control analysis of HLA-DRB1-DQB1 genotype from 2,555 multiple sclerosis patients and 1,365 healthy ethnically matched controls.

Genotype HLA-DRB1-DQB1	MS Patients	*%*	Controls	*%*	OR	95% C.I.		P*c*
*03∶01–*02∶01/*13∶03–*03∶01	40	1.6	5	0.4	4.3	1.7	11	2.2×10^−02^
*03∶01–*02∶01/*15∶01–*06∶02	50	2	7	0.5	3.9	1.8	8.6	9.0×10^−03^
*03∶01–*02∶01/*03∶01–*02∶01	339	13.3	65	4.8	3.1	2.3	4	2.0×10^−15^
*03∶01–*02–01/*04∶05–*03∶01	96	3.8	19	1.4	2.8	1.7	4.5	8.1×10^−04^
*04∶05–*03∶01/*11	54	2.1	12	0.9	2.4	1.3	4.6	1.2×10^−01^
*03∶01–*02∶01/*08	28	1.1	8	0.6	1.9	0.9	4.1	NS
*16∶01–*05∶02/*04∶05–*03∶01	51	2	15	1.1	1.8	1	3.3	NS
*03∶01–*02∶01/*04	180	7	70	5.1	1.4	1.1	1.9	5.4×10^−01^
*03∶01–*02∶01/*11	212	8.3	91	6.7	1.3	1	1.6	NS
*16∶01–*05∶02/*04	99	3.9	48	3.5	1.1	0.8	1.6	NS
*03∶01–*02∶01/*01	104	4.1	52	3.8	1.1	0.8	1.5	NS
*01/*11	64	2.5	33	2.4	1	0.7	1.6	NS
*01/*04	53	2.1	33	2.4	0.9	0.6	1.3	NS
*04/*07	36	1.4	21	1.5	0.9	0.5	1.6	NS
*04/*11	63	2.5	41	3	0.8	0.5	1.2	NS
*16∶01–*05∶02/*13	20	0.8	14	1	0.8	0.4	1.5	NS
*03∶01–*02∶01/*16∶01–*05∶02	153	6	122	8.9	0.6	0.5	0.8	1.6×10^−02^
*04/*04	22	0.9	20	1.5	0.6	0.3	1.1	NS
*16∶01–*05∶02/*11	88	3.4	83	6.1	0.6	0.4	0.7	3.3×10^−03^
*16∶01–*05∶02/*01	44	1.7	42	3.1	0.6	0.4	0.8	1.6×10^−01^
*04∶05–*03∶01/*04	16	0.6	15	1.1	0.6	0.3	1.2	NS
*16∶01–*05∶02/*07	28	1.1	25	1.8	0.6	0.3	1	NS
*11/*11	37	1.4	37	2.7	0.5	0.3	0.8	1.6×10^−01^
*03∶01–*02∶01/*07	35	1.4	35	2.6	0.5	0.3	0.8	2.0×10^−01^
*03∶01–*02∶01/*14∶01-4–*05∶031	16	0.6	22	1.6	0.4	0.2	0.7	7.6×10^−02^
*07/*11	15	0.6	22	1.6	0.4	0.2	0.7	4.4×10^−02^
*16∶01–*05∶02/*16∶01–*05∶02	24	0.9	47	3.4	0.3	0.2	0.4	6.0×10^−07^
*14∶01-4–*05∶031/*16∶01–*05∶02	7	0.3	19	1.4	0.2	0.1	0.5	1.1×10^−03^
	1974		1023					
Total	2555		1365					

Only genotypes represented in at least 1% of the sample were considered. Pc = P corrected for the 28 considered genotypes. NS = not significant.

Rare haplotypes belonging to the same haplogroup were grouped together: as *11 were designed *11∶01-02-03-04 -*03∶01, *11∶01-*03∶03-*05∶02 and *11∶04-*06∶03; as *07 were designed *07∶01- *02∶01 and *07∶01-*03∶03; as *13 were designed *13∶01-*06∶03-*03∶03, *13∶02-*05∶01-*05∶031-*06∶02-*06∶04-*06∶05-*06∶09, *13∶05-*03∶01 and *13∶16-*06∶04; as *04 were designed *04∶01-*03∶01-*03∶02, *04∶02-*03∶02, *04∶03– *03∶01-02-04-05, *04∶04-*03∶02-*04∶02, *04∶05-*02∶01, *04∶05-*03∶02, *04∶06-*03∶02, *04∶07-*03∶01 and *04∶08-*03∶01; as *08 were designed *08∶01-*04∶02, *08∶03-*03∶01 and *08∶04-*03∶01-*04∶02; as *01 were designed *01∶01 *05∶01, *01∶02-*05∶01 and *01∶03- *05∶01.

### Analysis of Interactions between Haplotypes

In order to establish whether the increased or decreased risk due to specific DRB1-DQB1 genotypes was due to positive or negative interactions between haplotypes, transmission/not transmission analysis of the haplotype inherited from the parent not carrying the risk haplotype (i.e. X/Y parent) was performed in both affected and healthy offspring from 961 trios families. Offspring were stratified according to presence or absence of the risk haplotype and the transmission of the second haplotype from the other parent not carrying the risk haplotype (X/Y parent) was assessed. The analysis was performed only in families where the haplotype in consideration was present in at least 250 heterozygous parents. The analysis was possible only for *03∶01-*02∶01 families. These families offspring were then stratified according to carriage of the *03∶01-*02∶01 haplotype in positive or negative, and the transmission of the other haplotype from the parent *03∶01-*02∶01-negative (one parent carrying X/Y, where X/Y not *03∶01-*02∶01) was examined, comparing in both categories (*03∶01-*02∶01 positive or negative) affected and unaffected offspring. In affected offspring, there were 269 receiving and 422 not receiving the *03∶01-*02∶01 haplotype. The previously associated haplotypes *04∶05-*03∶01, *13∶03-*03∶01, *15∶01-*06∶02 and *08 were over-transmitted in both *03∶01-*02∶01-positive and -negative groups; however, the extent of ORs were higher in the *03∶01-*02∶01 -positive than in –negative offspring, suggesting that the co-presence of two susceptible haplotype concurs in increasing predisposition to the disease. Similarly, in both groups *14∶01-4-*05∶031, *16∶01-*05∶02 and 15∶02-*06∶01 haplotypes showed similar trend of transmission, but they were under-transmitted more consistently in the *03∶01-*02∶01-positive group. The *13 haplotype showed an opposite trend, as it was under-transmitted in the positive (OR = 0.6) and over-transmitted in the negative group (OR = 2.1), with significant difference between the two categories (P = 1.7×10^−2^), thus suggesting an opposite effect of this haplotype according to the presence or absence of the *03∶01-*02∶01 haplotype. The supporting data are reported in Table 4.

**Table 4:** Transmission disequilibrium test of the not-transmitted parental haplotype in multiple sclerosis patients carrying (DR3+) or not carrying (DR3−) the HLA-DRB1*03∶01-*02∶01 haplotype.

	DR3+				DR3−				DR3+ and DR3−	
	*(269 MS*	*patients)*			*(422 MS*	*patients)*			*Subgroups*	*compared*
Haplotype	T	NT	p(<0,05)	OR	T	NT	p(<0,05)	OR	?2	p(<0,05)
*14∶01-4–*05∶031	3	8	1.3×10^−01^	0.4	23	26	6.6×10^−01^	0.9	1.4	2.3×10^−01^
*16∶01–*05∶02	30	61	3.6×10^−04^	0.4	101	148	8.9×10^−04^	0.6	1.6	2.0×10^−01^
*10∶01–*05∶01	10	8	6.3×10^−01^	1.3	15	24	1.4×10^−01^	0.6	1.5	2.3×10^−01^
*04∶05–*03∶01	35	12	4.5×10^−04^	3.2	55	22	1.0×10^−04^	2.6	0.1	7.1×10^−01^
*12∶01–*03∶01	5	5		1	13	17	4.6×10^−01^	0.8	0.1	7.1×10^−01^
*15∶02–*06∶01	0	10	1.4×10^−03^	0	5	16	1.5×10^−02^	0.3	2.8	9.2×10^−02^
*13∶03–*03∶01	17	2	4.6×10^−04^	9	17	6	2.1×10^−02^	2.9	1.6	2.0×10^−01^
*16∶02–*05∶02	2	2		1	1	4	1.8×10^−01^	0.2	0.9	3.4×10^−01^
*03∶01–*03∶01	0	0			1	2	5.6×10^−01^	0.5		NA
*15∶01–*06∶02	14	6	6.8×10^−02^	2.4	18	10	1.3×10^−01^	1.8	0.2	6.8×10^−01^
*01	23	35	9.5×10^−02^	0.6	66	79	2.5×10^−01^	0.8	0.6	4.5×10^−01^
*04	43	36	3.9×10^−01^	1.2	100	74	3.4×10^−02^	1.4	0.2	6.5×10^−01^
*07	10	17	1.7×10^−01^	0.6	57	58	9.2×10^−01^	1	1.4	2.4×10^−01^
*08	9	1	1.1×10^−02^	9.3	14	8	2.0×10^−01^	1.8	2.4	1.2×10^−01^
*11	55	50	5.9×10^−01^	1.1	110	116	6.6×10^−01^	0.9	0.4	5.3×10^−01^
*13	8	14	1.9×10^−01^	0.6	29	14	2.0×10^−02^	2.1	5.7	1.7×10^−02^
*15	5	2	2.5×10^−01^	2.5	9	10	8.2×10^−01^	0.9	1.2	2.8×10^−01^
	269	269			634	634				

NA = no mating of this type was available.

Rare haplotypes belonging to the same haplogroup were grouped together: as *11 were designed *11∶01-02-03-04 -*03∶01, *11∶01-*03∶03-*05∶02 and *11∶04-*06∶03; as *07 were designed *07∶01- *02∶01 and *07∶01-*03∶03; as *13 were designed *13∶01-*06∶03-*03∶03, *13∶02-*05∶01-*05∶031-*06∶02-*06∶04-*06∶05-*06∶09, *13∶05-*03∶01 and *13∶16-DQB1*06∶04; as *04 were designed *04∶01-*03∶01-*03∶02, *04∶02-*03∶02, *04∶03– *03∶01-02-04-05, *04∶04-*03∶02-*04∶02, *04∶05-*02∶01, *04∶05-*03∶02, *04∶06-*03∶02, *04∶07-*03∶01 and *04∶08-*03∶01; as *15 were designed *15∶01-*05∶01-02 and *15∶01-*06∶01-03; as *08 were designed *08∶01-*03∶01-*04∶02, *08∶03-*03∶01 and *08∶04-*03∶01-*04∶02; as *01 were designed *01∶01 *05∶01, *01∶02-*05∶01 and *01∶03- *05∶01.

To control whether these findings were due to an effect of population (i.e., whether the over- or under-transmission was casual) the analysis was repeated in unaffected offspring. There were 134 individuals receiving and 395 not receiving the *03∶01-*02∶01 haplotype. No significant differences were found in both categories of *03∶01-*02∶01-positive individuals and –negative individuals (data not shown). Interaction between haplotypes was also examined using logistic regression analysis. In the model status of individual (affected/non affected) was considered as dependent variable, while all haplotypes used in the case-control analysis were considered as independent variables. The dependent variable was considered in relation to the independent variables and the second order interactions between them. Significant interactions between *03∶01-*02∶01 and *14∶01-4-*05∶031 (OR = 0.26, 95% CI 0.12–0.58, P = 1.0×10^−3^), *03∶01-*02∶01 and *16∶01-*05∶02 (OR = 0.51, 95% CI 0.37–0.72, P = 1.0×10^−4^), *03∶01-*02∶01 and *07 (OR = 0.36, 95% CI 0.20–064, P = 5.0×10^−4^), *14∶01-4-*05∶031 and *16∶01-*05∶02 (OR = 0.31, 95% CI 0.11–0.84, P = 2.0×10^−^2), *13∶03-*03∶01 and *11 (OR = 0.19, 95% CI 0.05–0.69, P = 1.0×10^−2^), *1 and *07 (OR = 2.61, 95% CI 1.08–6.29, P = 3.0×10^−2^) and *01 and *11 (OR = 2.08, 95% CI 1.27–3.40, P = 4.0×10^−3^) were observed. According the these interactions, risk was significantly lowered in the *03∶01-*02∶01/*14∶01-4-*05∶031 (OR = 0.43, 95% CI 0.22–0.87, P = 2.0×10^−2^) and in the 14∶01-4-*05∶031/*16∶01-*05∶02 (OR = 0.19, 95% CI 0.08–0.46, P = 3.0×10^−4^) genotypes. Data are reported in Table S1.

### Mathematical Model of Interaction

We explored a simple mathematical model of interactions in order to determine whether the risk carried by the genotypes was different from the sum of the risk of the two haplotype (ORha and ORhb). The idea of the model is based on the hypothesis of statistical independence between ORha and ORhb. If this is true, their log-values can be combined as sum, giving in this way the global protective or predisposing character of the genotype as a simple balance between the character of the single haplotype OR. This would lead to the introduction of the “expected” overall OR to compare with the “observed”, or measured, OR.

Considering the equation:
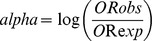



The alpha parameter can be used as a “probe” for the violation of this composition law.

In Table 5 are reported the acquired data: the haplotypes considered and the resulting genotype, the OR values for the haplotypes (*ORha* and *ORhb*) and for the related genotypes (*ORgobs*), the expected genotypic OR values (*ORgexp*) for a pure additivity law of composition of haplotypes ORs and the *alpha*. If *ORgobs<ORgexp* it means that the coupling creates a protective effect (decreasing the expected risk); on the contrary, for *ORgobs>ORgexp* there is a predisposing effect of the haplotypes coupling. The second column in Table 5 shows the predisposing or protective absolute character of haplotypes examined on the basis of the respective *ORha* and *ORhb*.

**Table 5:** HLA-DRB1-DQB1 genotypes from 2,555 multiple sclerosis patients, protective-predisposing nature for haplotypes in the second column, OR values of the individual haplotype (ORha and ORhb), the genotypic value of OR expected under log-additivity model (ORgexp) and the observed one (ORgobs), values of alpha parameter of the additivity violation.

Genotype HLA-DRB1-DQB1	Haplotypes character	ORha	ORhb	ORgobs	ORgexp (additivity)	Alpha
*16∶01-*05∶02/*14∶01-4-*05∶031	protective/protective	0.6	0.5	0.2	0.3	−0.18
*16∶01-*05∶02/*16∶01-*05∶02	protective/protective	0.6	0.6	0.3	0.36	−0.08
*07/*11	protective/protective	0.7	0.8	0.4	0.56	−0.15
*16∶01-*05∶02/*03∶01-02∶01	protective/predisposing	0.6	1.7	0.6	1.2	−0.23
*03∶01-*02∶01/*04∶05-*03∶01	predisposing/predisposing	1.7	2.1	2.8	3.57	−0.11
*03∶01-*02∶01/*03∶01-*03∶01	predisposing/predisposing	1.7	1.7	3.1	2.89	0.03
*03∶01-*02∶01/*15∶01-*06∶02	predisposing/predisposing	1.7	2	3.9	3.4	0.06
*03∶01-*02∶01/*13∶03-*03∶01	predisposing/predisposing	1.7	3.3	4.3	5.61	−0.12

Rare haplotypes belonging to the same haplogroup were grouped together: as *11 were designed *11∶01-02-03-04 -*03∶01, *11∶01-*03∶03-*05∶02 and *11∶04-*06∶03; as *07 were designed *07∶01- *02∶01 and *07∶01-*03∶03.

Two kind of interaction between haplotypes can be depicted: i) a neutral interaction, in which the global risk is coherent with the sum of the single haplotype risks (gene-dosage effect); ii) a negative interaction, in which the genotypic OR observed is lower than the sum of OR of the two haplotypes. We have defined a sort of empirical ranking of the interaction strength according to the α value:

the α values which lie between the range 0.01 and 0.09 correspond to no interaction (neutral interaction), while for values of α above or below the range there is interaction and the magnitude of such interaction being as great as the α value is distant from the range.

In Table 5 we summarize these findings about the observed interactions. Interactions of the predisposing allele *03∶01 with itself or with the other predisposing *15∶01 and *04∶05 haplotypes are found to be neutral, while all other interactions were negative.

### Sequence and Alignment Analysis

The sequence of the eight associated HLA-DRB1 alleles, namely *16∶01, *14∶01, *15∶02, *04∶05, *13∶03, *03∶01, *15∶01 and *08∶01 were also analyzed. The *11 was excluded from the analysis because it was not confirmed to be associated by the RPE analysis. The results are reported in Table 6, showing only the positions with a residue variation between the allele.

**Table 6:** Sequence alignment of the eight MS associated DRB1 alleles.

Pos	9	10	11	12	13	26	32	33	37	47	57	60	67	70	71	73	74	77	86	96	98	104	112	120	133	140	142	149
*16∶01	W	Q	P	K	R	F	Y	N	S	Y	D	Y	F	D	R	A	A	T	G	Q	K	S	H	S	L	A	M	Q
*15∶02	W	Q	P	K	R	F	Y	N	S	F	D	Y	I	Q	A	A	A	T	G	Q	K	S	H	S	L	A	M	Q
*14∶01	E	Y	S	T	S	F	H	N	F	Y	A	H	L	R	R	A	E	T	V	H	K	S	Y	S	R	T	V	H
*04∶05	E	Q	V	K	H	F	Y	H	Y	Y	S	Y	L	Q	R	A	A	T	G	Y	E	A	H	N	R	T	V	Q
*13∶03	E	Y	S	T	S	F	Y	N	Y	Y	S	Y	I	D	K	A	A	T	G	H	K	S	H	S	R	T	V	H
*03∶01	E	Y	S	T	S	Y	H	N	N	F	D	Y	L	Q	K	G	R	N	V	H	K	S	H	S	R	T	V	H
*15∶01	W	Q	P	K	R	F	Y	N	S	F	D	Y	I	Q	A	A	A	T	V	Q	K	S	H	S	L	A	M	Q
*08∶01	E	Y	S	T	G	F	Y	N	Y	Y	S	Y	F	D	R	A	L	T	V	H	K	S	H	S	R	T	V	H
**Pock**	**9**		**6**		**46**					**7**	**9**		**7**	**4**	**47**		**4**		**1**									

Only positions with different residues are considered. The first line reports the residue position and the last line the pocket or pockets to which it belongs.

**Figure 1:**
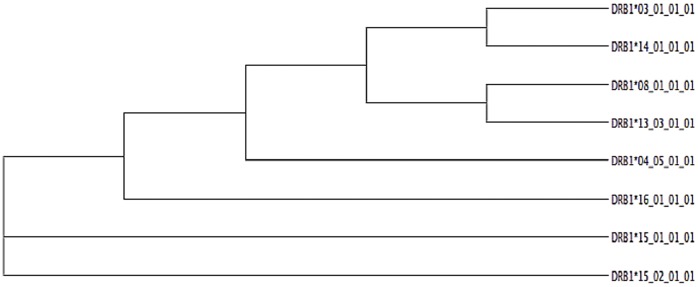
Phylogenetic tree of the MS associate DRB1 alleles.

The phylogenetic tree of these alleles is shown in Figure 1.

It is immediate to extract two main allele clusters: the DR2 group (*16∶01, *15∶02, *15∶01) that was recently analyzed [17], and the new cluster represented by *14∶01, *04∶05, *13∶03, *08∶01 and *03∶01. This grouping can be also understood at a glance observing the sequence alignments in Table 6, where position 9 (W or E) and position 133 (L or R) immediately distinguish between the two groups. Importantly, the same result still holds when restricting the cluster analysis to the allele positions that belong to a known anchoring pocket.

As done previously for the DR2 group [17] the possible functional aspects involved in the allele molecular antigen presentation mechanisms was hypothesized. Starting from the sequence alignment (Table 6), it was observed that the main differences between *14∶01 and *03∶01 are five residues at position 47, 57, 70 71, 74, which belong to anchoring pockets 4, 7 and 9. Subsequently, it was noted that the most important changes occur at position 74 (pocket 4, a negatively charged residue in *14∶01 and a positively charged one in *03∶01), 70 (pocket 4, a positive residue in *14∶01 and an hydrophilic one in *03∶01) and position 57 (pocket 9, small hydrophobic residue in *14∶01 and negative residue in *03∶01). Thus, as expected, the most striking differences between *03∶01 and *14∶01 alleles are observed in the binding region, involved in antigen presentation.

**Figure 2:**
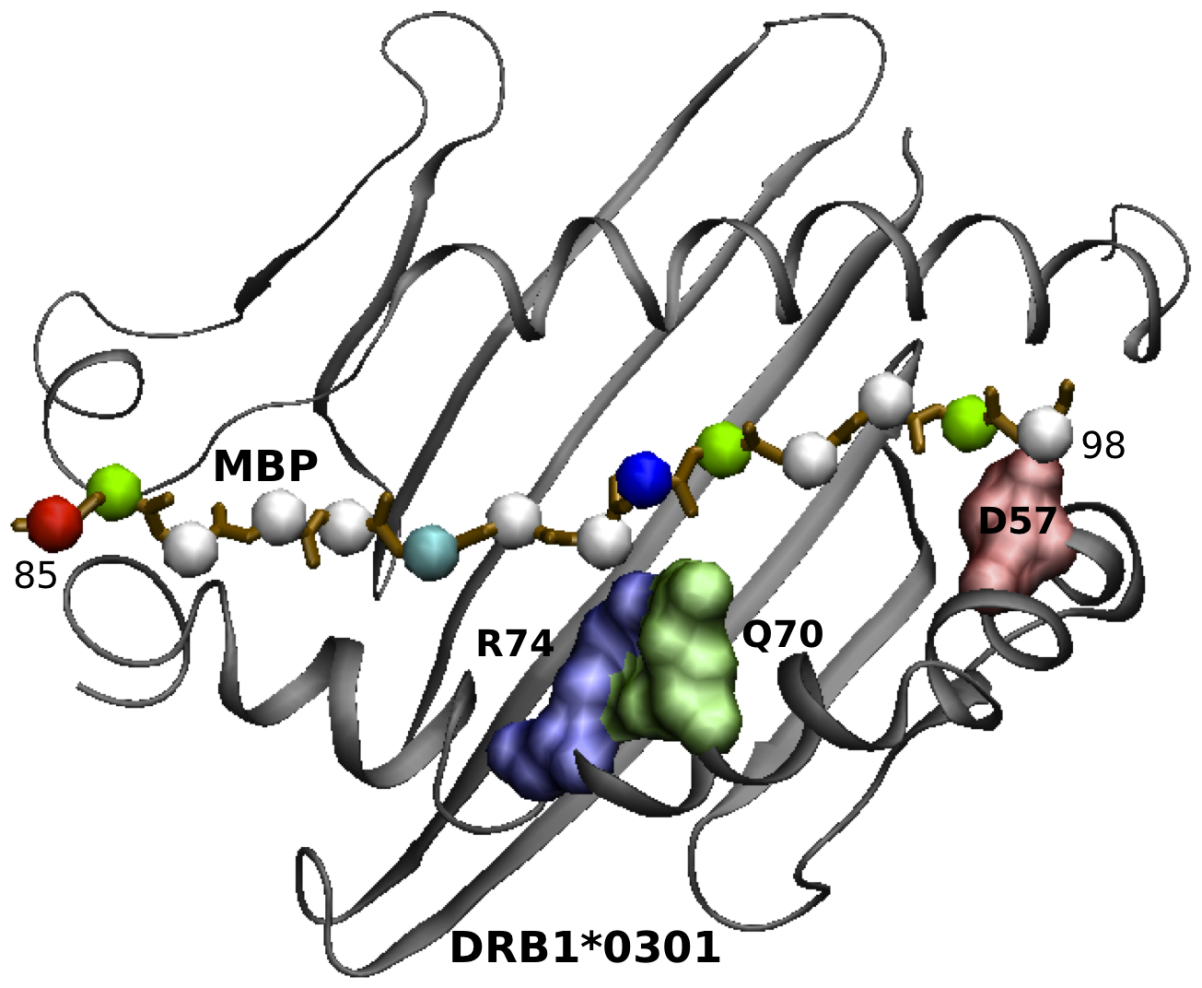
Binding region for the MHC-peptide complex for DRB1*03∶01 allele. MHC binding region is shown in cartoon representation (black), MBP peptide backbone is shown in ball-stick representation. The residues in pocket P4, and P9 are shown in surface representation and are colored based on residue type (blue: basic, red: acidic, green: polar).

**Figure 3:**
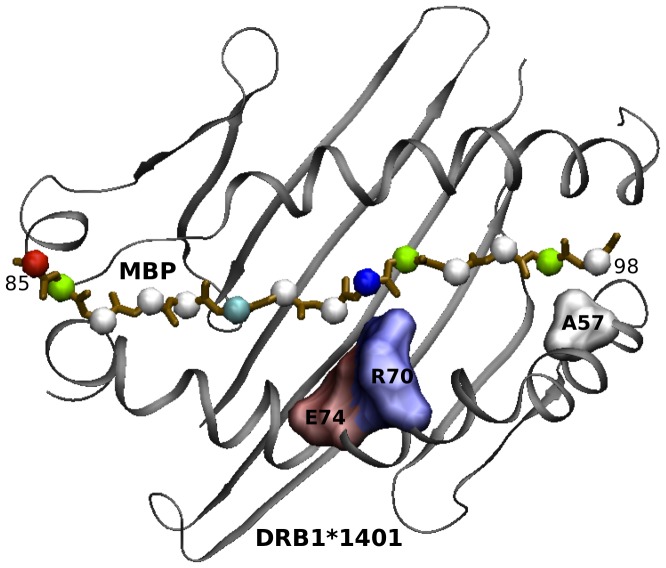
Binding region for the MHC-peptide complex for DRB1*14∶01 allele. MHC is shown in cartoon representation (black), MBP peptide backbone is shown in ball-stick representation. The residues in pocket P4, and P9 are shown in surface representation and are colored based on residue type (blue: basic, red: acidic, green: polar).

**Figure 4:**
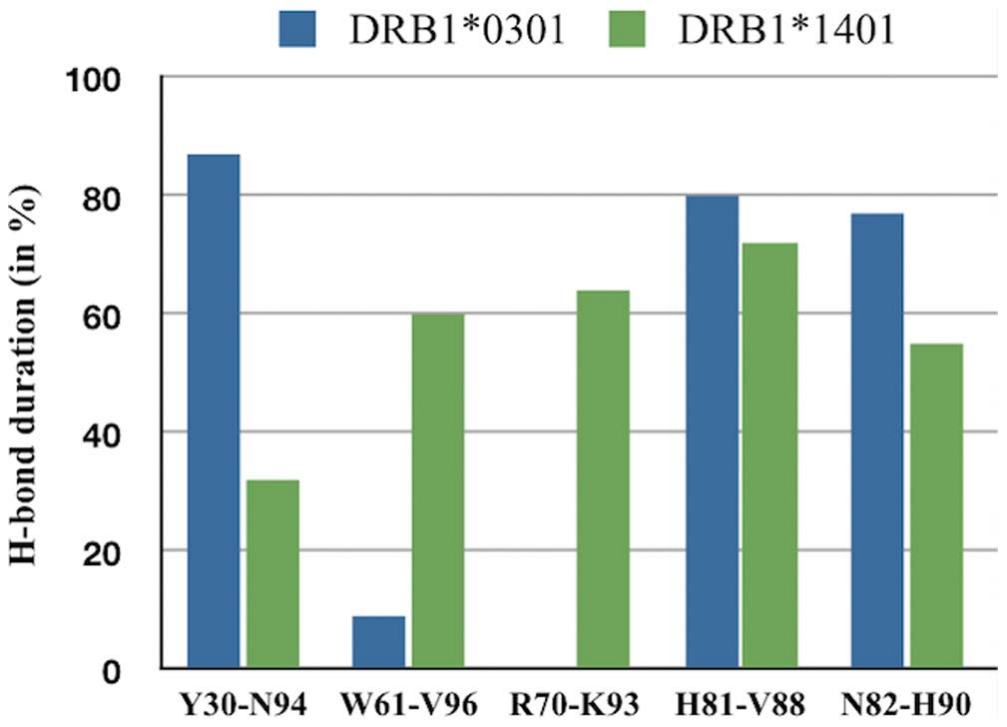
MBP-MHC H-bonds. Percentual duration time of MBP-established H-bond, during 3 ns MD simulation, for the residues of DRB1*03∶01 (in blue) and DRB1*14∶01 (green) binding site.

Subsequently a short molecular dynamics (MD) simulation of 3 ns for both alleles loaded with MBP 85–99 peptide was performed. For both alleles, we generated an average structure after 3 ns of MD simulation (Figure 2–3) that was used to highlight structural differences at pockets P4, P9. The first focus was the analysis of the stable (i.e. present at least for 10% of the simulation time) H-bonds established between amino acids in the binding site and those belonging to the self peptide. On scanning all possible amino acid pair interactions with MBP, it is interesting to note that the most relevant divergence between the two alleles is that at position 70, where only *14∶01 is capable to form a durable H-bond with K93 of MBP (Figure 4).

Concerning the other alleles in the new group, it was noticed that *13∶03 has a charged residue at position 70, as *14∶01 although with reversed polarity (D and R respectively), while both *03∶01 and *04∶05 have an hydrophilic residue (Q), but *04∶05 displays a small nonpolar residue (A) in position 74 with respect to the positively charged one (R) for *03∶01. These difference can thus highlight a distinct global binding characteristics, due to the whole pocket 4 polar environment, between *03∶01 and *04∶05. DRB1*08∶01 shares an high sequence identity with the *13∶03 allele, with small structural differences located at position 74 and 86, where in both cases was observed a small non-polar hydrophobic residue in *13∶03 and an hydrophobic one in *08∶01.

**Figure 5:**
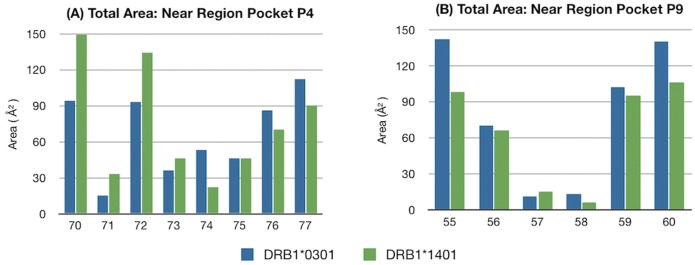
Area calculation near Pockets. Total available area (in unit Å^2^) near (left histogram) the P4 region (right histogram) P9 region for the alleles DRB1*03∶01 (in blue) and DRB1*14∶01 (green), in the absence of MBP peptide.

**Figure 6:**
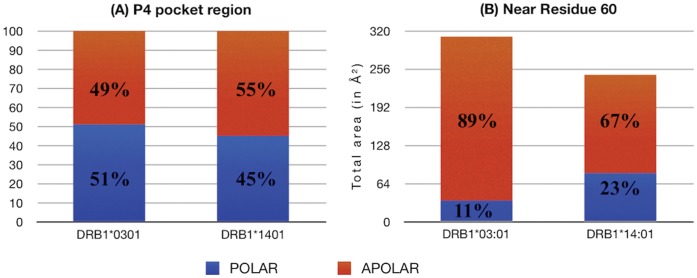
Polar and apolar area calculation. In percentage, polar and apolar area available near (A) P4 region and (B) residue 60 (close to pocket P9 region), for the alleles DRB1*03∶01 and DRB1*14∶01 respectively.

Our previous sequence analysis identified also pocket 9 as significant position which distinguishes the alleles; therefore we have subsequently investigated the characteristics for the two alleles, *03∶01 and *14∶01, in both the regions near pocket P4 (Figure 5-left) and P9 (see Figure 5-right) with respect to the pocket surrounding area that is available for binding. This comparison showed a significant difference between the two alleles only for pocket 4, particularly at position 70, 72 and 74 (Figure 5-left). Summing the total area available near P4 pocket region, a significant increase (590 Å^2^) for *14∶01 allele with respect to *03∶01 one (535 Å^2^) was noted that it is mainly due to residue number 70, 72, as can be seen from Figure 5-left. Subsequently the polar/apolar area ratio available near the P4 region for both alleles (Figure 6) was studied, obtaining 51∶49 for *03∶01 and 45∶55 for *14∶01, once again highlighting a distinct global binding capability at pocket 4 for the two alleles.

## Discussion

The strong association between HLA-DRB1-DQB1 loci and MS has been established across many populations, with consistent findings indicating that predisposition is carried by the *15∶01-*06∶02 haplotype in all populations of North-European ancestry [4], while in Israel [8] and in Mediterranean [5]–[7] populations predisposition to the disease is carried by different DRB1 variants. A recent genome-wide association study confirmed the *15∶01 allele as the strongest genetic determinant in MS and, after conditioning to the *15∶01, an association with the *03∶01 and the *13∶03 allele emerged [4].

Several studies [9]–[12] have indicated the presence of alleles which confers resistance to the disease and modulates the permissive effect of the *15∶01 allele, thus suggesting that the autoimmune response may be lowered or cancelled by the co-presence of both susceptible and protective alleles. Indeed, two copies of the *15∶01 allele determined the highest risk [9], while the *15/*14 genotype considerably lowered the risk of the disease [9]–[12], thus supporting a disease association gradient due to complex interactions among DRB1 alleles.

In Sardinia, an Italian island having a very high prevalence of MS [13], [14] and a peculiar genetic background [15], a heterogeneous HLA association with MS has been reported [16]. Recently, we have re-analysed the risk carried from HLA class II variants, and found it to be specifically determined by both DRB1 and DQB1 alleles (DRB1-DQB1 haplotype) in Sardinian MS patients, thereby confirming the haplotype association, establishing the independence of the associated haplotypes and assessing the genotypic risk [17].

In particular, the independence of the positively associated *13∶03-*03∶01, *04∶05-*03∶01 and *03∶01-*02∶01 haplotypes was established and we found that these predisposing haplotypes are inherited according to a dominant model, while the protective *16∶01-*05∶02 haplotype was inherited in a recessive way. These data delineated a model constituted by a dominantly acting susceptibility gene contained on, or near to, the *04∶05-*03∶01, *13∶03-*03∶01, *03∶01-*02∶01 haplotypes, in conjunction with the absence of a protective gene required for the maintenance of peripheral tolerance.

In the present study we have analyzed the presence of interactions between DRB1-DQB1 predisposing and protective haplotypes. For this purpose, we firstly determined haplotypic ORs in more than 2,500 patients. To be sure that the different haplotypes associated with MS were not due to a displacement effect, data were confirmed using the RPE method. Case-control analysis of the associated haplotypes confirmed the well-known predisposing haplotypes *13∶03-*03∶01, *04∶05-*03∶01, *15∶01-*06∶02 and *03∶01-*02∶01 and the already reported protective *16∶01-*05∶02 and *15∶02-*06∶01 haplotypes [17], but revealed two other protective haplotype: *11 and *14∶01-4-*05∶031; however, the *11 haplotype was not confirmed using the RPE method. It is of interest to note that the *14 and *11 alleles have been reported as a protective ones either in a study from European [9], [19] or from Canadian MS population [10]; in addition, both molecules interact with the major North-European genetic MS determinant, the *15∶01 allele, mitigating its risk. In the same cohort of patients and controls, genotypic ORs were established. Two typologies of interaction between haplotypes can be depicted: i) a neutral interaction, in which the global risk is coherent with the sum of the single haplotype risks (gene-dosage effect); ii) a negative interaction, in which the co-presence of two haplotypes resulted in a risk lower than the sum of the single haplotypes. Thus, interactions of the predisposing allele *03∶01 with itself or with the other predisposing *15∶01and *04∶05 haplotypes are found to be neutral interactions. In all these cases, the ORs of genotypes demonstrate an additive interaction and the *alpha* parameter is comprise between 0.01 and 0.09. All other combinations of haplotypes reported in Table 5 showed negative interactions. Indeed, the effect of the genotypes expressed as a global OR decreases the risk expressed by the ORs of the corresponding haplotypes. In all these cases, the effect of interactions is not in line with the additive model and the stronger is the interaction, the higher is the deviation of *alpha* from the range of 0.01 and 0.09. The interactions here described are in agreement with a model in which molecular structure of DRB1-DQB1 alleles constituting the genotype modulates the MS risk through a synergic action of molecules permissive or not-permissive for the same or different MBP epitopes or perhaps for different autoantigens.

We cannot exclude that the observed protective effects between different DRB1-DQB1 haplotypes could be due to linkage-disequilibrium with HLA class I alleles. A protective effect of the HLA-A*02 allele, independent from the HLA-DRB1*15∶01 allele, has been established by several studies [4], [20]-[22]; in addition to the effect of the HLA-A and HLA-DRB1 loci, also the HLA-B*12 allele has been suggested to influence MS risk [22]. Considering the peculiar class II gene-based substructure of Sardinian population, to know whether class I alleles influence predisposition and protection to MS in Sardinian patients as in other European population [4], [20]–[22] can be relevant to understand molecular mechanisms underlying the disease’s pathogenesis.

As observed from phylogenetic tree analysis, two main allele clusters are evident: the DR2 group and the new cluster represented by *14∶01, *04∶05, *13∶03, *08∶01 and *03∶01. As observed, sequence alignment showed that the two groups of alleles are distinguished by different residues at positions 9 (W or E) and 133 (L or R). Indeed, as already described [17], the variable residue at position 86 and position 38 of the DRB1 chain are the only one that differentiated between the protective *16∶01 and *15∶02 from the predisposing *15∶01 DR2 alleles. In the case of the second group, the most important changes occur at position 74 (pocket 4) and 57 (pocket 9), which differentiated the protective *14∶01 and the predisposing *03∶01 alleles.

Barcellos et. al [11], have previously noted DRB1*14∶01 to be an unique allele in having a basic residue Histidine (H) at position 60 (close to pocket 9), while the other seven alleles (see Table 6) share an aromatic residue Tyrosine (Y). The authors hypothesized this specificity could impact the pocket 9 shape and binding ability, leading to a sub-optimal docking of encephalitogenic peptides, conferring protection over *15∶01. In our present study, we went a step further and quantify this difference by evaluating the total accessible area near residue 60 (considering residues 59, 60 and 61), for alleles *03∶01 and *14∶01, as shown in Figure 6B. Interestingly, we note a significant difference between the two alleles (*03∶01, *14∶01) considering both the total area (301 Å^2^, 246 Å^2^) and also the polar (11%, 23%) and apolar area (89%, 67%) ratio. Furthermore, we observed another unique characteristics, this time of *03∶01 at position 57 (pocket 9), with a negatively charged aminoacid (D), while all the other alleles in the new group show a small hydrophobic residue (A or S). Altogether, our study confirms the importance of pocket 9 for characterizing the alleles with respect to the disease association. Nevertheless, in our case the pocket 4 proved to be more relevant than 9 to functionally distinguish the alleles in the new cluster, particularly the protective *14∶01 and the predisposing *03∶01. We can thus speculate that both pocket 4 and 9 should act synergistically to confer a specific binding patterns of relevant epitopes, specifically conferring resistance to the allele able to bind its ligand at pocket 9 weaker than and at pocket 4 stronger than the susceptibility alleles. This is in line with the pocket role of global and specific anchoring [23], respectively, and on the complex immunological picture where higher binding affinities of the HLA for the peptide do not immediately lead to an higher affinity for the TCR, nor even to an higher triggering capability of T cells. The dynamical aspects of the peptide behavior inside the whole binding site and the presence of externally exposed bumps are likely to have a deeper impact on TCR recognition.

MD simulation of *14∶01 and *03∶01 loaded with MBP 85–99 peptide supports and enforces our preceding findings, based on a simplistic residue comparison. Interestingly, Wucherpfennig and Strominger [24] suggested that MBP residues K93, F91, and H90 are primary TCR contact points. Therefore, we confirm that *14∶01 allele is showing a quite distinct capability to anchor the MBP peptide in pocket 4, with respect to *03∶01, particularly for residue K93, likely impacting on TCR recognition and ultimately in T cell triggering and activation. From these findings, we preliminary conclude that, while *14∶01 (protective) and *03∶01 (predisposing) are the two closest alleles in the group from the phylogenetic (and thus sequence identity) point of view, and they show striking differences in binding MBP 85–99 peptide in pocket 4 at position 70.

Concerning the other alleles in the new group, the *13∶03, *08∶01 and *04∶05 predisposing alleles, the first two have a charged residue at position 70, as *14∶01 although with reversed polarity (D and R respectively). Further, both *03∶01 and *04∶05 have an hydrophilic residue (Q) at position 74, while *04∶05 and *08∶01 display an hydrophobic residue (A or L, respectively), and *03∶01 a positively charged one (R). These differences can thus highlight distinct global binding capabilities, due to the whole pocket 4 polar environment, between *03∶01 and *04∶05.

It is important to note that DRB1 position 70–74 is also the region object of the shared epitope hypothesis and its connection with some autoimmune diseases, particularly rheumatoid arthritis (RA) has been reported [25]. A further restriction for functional hypothesis of disease mechanisms comes considering the hot spots for TCR recognition of Pocket 4, namely position 70, 71 and 74 [26]. For instance, some authors have found that a specific amino acid pattern at position 70, 71 and 74 (Q or R, R or K, A, respectively, as in *04∶05, showing a tendency to a more positively charged pocket) were predisposing for RA, while other motifs were protective to different degrees or neutral [27]. More recently, a glutamic acid (E, negatively charged) at position 71 or 74 was associated to the clinical course of MS [28], namely it was found more present in PP patients than RR or SP ones. Other authors have reported a MS association to alanine (A, hydrophobic) at position 71 [29]. Our analysis thus confirms the importance of pocket 4, and in our case particularly of position 70.

Together, these data can suggest that propensity to MS observed in Sardinian population can be due to a complex presence of various HLA-DRB1-DQB1 molecules, each provided with different affinity and possible functional peculiarity in the range of antigen(s) presentation. The models we generated included only the MBP 85–99 peptide, but we are in progress to perform molecular dynamics simulation with other peptides, including exogenous peptides from pathogens as Epstein-Barr virus and Mycobacterium avium paratuberculosis, very common in the island and recently involved in MS pathogenesis [30], [31].

## Subjects and Methods

### Patients

We examined 2,555 MS patients, 961 of which coming from families consisting of one affected sibling and both healthy parents (trios), 331 healthy siblings (one from each family) of patients coming from the same families, and 1,365 healthy ethnically matched controls. All patients participating in the study attended the MS Clinic at University of Cagliari (Italy). The study was conducted in accordance with the Helsinki Declaration and approved by University of Cagliari/ASL8 (Italy) ethics committee. All subjects gave informed written consent.

All patients included in the study met MS criteria [32], [33]. The study cohort included healthy subjects and Sardinian MS patients, many of them had a Sardinian ancestry of three generations or more. The sample included in the study was representative of the total Sardinian MS population consisting in about half of the estimated Sardinian population with MS (the actual Sardinian population is about 1 million and 550,000 inhabitants). Subjects included in the study came from each Sardinian province, and were present in proportion to the number of inhabitants of each province.

### Genotyping

Typing of the HLA-DRB1* and -DQB1* loci was performed as described previously [16]. Briefly, the polymorphic second exon of the HLA-DRB1* and -DQB1* genes was amplified and the amplified products were subjected to dot-blot analysis using primers and SSO probes as described previously [16]. A total of 4,788 individuals were fully typed with high resolution typing. The DRB1-DQB1 haplotypes reported in MS cases and controls were assigned following the known pattern of linkage disequilibrium in Caucasians and Sardinians [34], [35]. In the case of rare associations, the haplotypes were accepted only when the haplotype present on the other chromosome was well defined. Ambiguous assignments were resolved by excluding those individuals. Moreover, 943 haplotypes were established following the co-segregation in 943 MS trio families and was assessed by the TDT phase program (version 2.403), as reported [17]. Only certain haplotypes from parental genotype data, and in absence of intercrosses (that is when both parents were heterozygous for the same alleles), were considered in the analysis.

Rare haplotypes belonging to the same haplogroup were grouped together. Thus, as *11 were designed *11∶01-02-03-04 -*03∶01 (case 12.56%, control 14.74%), *11∶01-*03∶03-*05∶02 (case 0.21%, control 0.47%) and *11∶04-*06∶03 (case 0.06%, control 0.15%); as *07 were designed *07∶01- *02∶01 (case 3.35%, control 4.29%) and *07∶01-*03∶03 (case 0.50%, control 0.95%); as *13 were designed *13∶01-*06∶03-*03∶03 (case 1.13%, control 1.31%), *13∶02-*05∶01-*05∶031-*06∶02-*06∶04-*06∶05-*06∶09 (case 0.95%, control 1.02%), *13∶05-*03∶01 (case 0.07%, control 0.07%) and *13∶16- *06∶04 (case 0.00%, control 0.03%); as *04 were designed *04∶01-*03∶01-*03∶02 (case 0.10%, control 0.11%), *04∶02-*03∶02 (case 1.46%, control 1.06%), *04∶03– *03∶01-02-04-05 (case 3.50%, control 4.32%), *04∶04-*03∶02-*04∶02 (case 0.23%, control 0.29%), *04∶05-*02∶01 (case 1.07%, control 1.46%), *04∶05-*03∶02 (case 4.67%, control 3.87%), *04∶06-*03∶02 (case 0.01%, control 0.00%), *04∶07-*03∶01 (case 0.13%, control 0.18%) and *04∶08-*03∶01 (case 0.01%, control 0.00%); as *15 were designed *15∶01-*05∶01-02 (case 0.56%-control 0.47%) and *15∶01-*06∶01-03 (case 0.27%, control 0.25%); as *08 were designed *08∶01-*03∶01-*04∶02 (case 1.04%, control 0.51%), *08∶03-*03∶01 (case 0.04%, control 0.00%) and *08∶04-*03∶01-*04∶02 (case 0.23%, control 0.25%); as *01 were designed *01∶01 *05∶01 (case 4.24%, control 4.54%), *01∶02-*05∶01 (case 2.75%, control 3.95%) and *01∶03- *05∶01 (case 0.15%, control 0.10%).

### Statistical Analysis

In the case-control analysis a highly conservative Bonferroni correction to P values (P_c_) for the total number of haplotypes (N = 15) or genotypes (N = 28) considered in the analysis was applied.

### The Relative Predispositional Effect Method

The RPE method [18] sequentially compares allele frequencies in patients and controls to determine their predisposition, protective, or neutral effects relative to each other. The overall frequency distribution of all haplotypes and genotypes at the DRB1-DQB1 loci was compared with the distribution in controls by using a χ2 test to detect significant deviations. To identify the haplotype with the greatest predispositional effect, the individual haplotype was reviewed for their contribution to the overall χ2 value. The frequencies in patients and controls were compared using the normal distribution (Z statistic). The procedure was repeated to find the next largest RPE, but haplotype detected in the previous round was excluded in both patients and controls and the expected frequency distribution of the remaining haplotypes in the controls was normalized accordingly. This process was sequentially continued until no significant overall deviation between patients and controls was observed.

### Logistic Regression Analysis

Interaction between haplotypes was also examined using logistic regression analysis. In the model status of individual (affected/non affected) was considered as dependent variable, while all haplotypes used in the case-control analysis were considered as independent variables. The dependent variable was considered in relation to the independent variables and the second order interactions between them.

### Mathematical Model of Interaction

The expected OR of predisposing and protective genotypes can be evaluated from the ORs of the single haplotypes with the assumption of statistical independence, that is the frequency of the genotype is given by the product of the frequencies of the two haplotypes. In this case, it is straightforward to obtain a rather complex model that can be simplified if, as in our cases, the haplotypic ORs are reasonably close to the unity. We then propose a simple empirical model linking haplotypic ORs to the expected genotypic OR, allowing the evaluation of the differences with respected to the really observed genotypic OR. In the model we considered only the significantly associated genotypes showed in Table 3.

Given two haplotypes *ha* and *hb*, and their respective odds ratio *ORha* and *ORhb*, their global effect in the case of independent risk combination will provide an expected genotypic OR (*ORgexp)* equals to the two haplotypic ORs product:
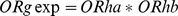



This can be immediately translated to an additivity property in logarithmic scale:
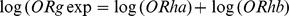



In fact, the logarithm of the OR has been shown to possess a simpler statistical behavior than the OR itself, particularly allowing a better relative risk assessment and ORs comparison. The first point is that the logarithm introduces a symmetry respect to the grouping adopted to evaluate the ORs. Assume for instance that in a case OR = 1/4 and in another case OR = 4. These two cases are of course exactly specular, but the OR does not show such an explicit symmetry and, more importantly, its statistical properties are not symmetrical. On the other hands, taking the logarithm, we get −1.39 and +1.39 respectively, thus indicating in an effective way the specular nature of the two hypothetical cases. Moreover the OR tends to amplify the relative risk probability, and this effect is tempered by the use of the logarithm. Consider for instance the following two odds: 8∶2 (80% risk) and 4∶6 (40% risk). The relative risk is just 2, while the ORs ratio is 6 and its log is 1.79 (closer to the relative risk than the OR).

Without entering into the details, the statistical significance of the introduced *alpha* parameter is linked to the *p-*values of the genotypic OR (Table 3) and of the single haplotypic ORs (Table 1), and requires the use of the full model of which the *log*-additive one is just an approximation. For our purposes, it is sufficient to state that in our simplified model the significance ranking of the observed genotypic ORs is preserved.

We can now formally introduce the deviation of the observed genotypic OR (*ORgobs*) with respect to the expected one, through an empirical parameter *alpha*:
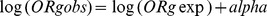



or
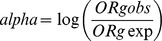



If *alpha* is included between 0.01 and 0.09, then there is no deviation and the observed disease risk follows a pure additivity composition of the two single haplotypic risks. If *alpha* differs from the range of 0.01 and 0.09 there is a deviation, represented by a violation of the simple additivity, eventually meaning that the two different haplotypes interact in a non-linear way to shape the global disease’s risk. Specifically, if *alpha* >0 the observed OR is higher than expected, thus showing a global predisposing effect; if *alpha* <0 the observed OR is lower than observed, thus showing a global protective effect.

### Sequence and Phylogenetic Analysis

The associated DRB1 allele sequences were retrieved in the NCBI dbMHC database [36] and preliminarily aligned by standard blastp tools [37]. Phylogenetic tree of the alleles was generated using the Clustal W–phylogeny tool [38], and visualized with the software Dendroscope [39].

### Molecular Dynamics Simulation

The starting structure for *03∶01 was taken from x-ray structure of *03∶01-CLIP complex (pdb id: 1A6A), and the structure for *14∶01 was homology modeled using the *03∶01 as template. The structure for self-peptide MBP was taken for *15∶01-MBP (pdb id: 1BX2) complex crystallographic structure. The MBP-HLA complex for both alleles *03∶01 and *14∶01 were placed alternatively in water-box and counter-ions were added to neutralize the system. In total each complex system consisted of 50.000 atoms. We used Amber force-field parameters [40] for the complex and TIP3P parameters for the water molecules. Long-range electrostatic interactions were evaluated using particle mesh Ewald with a [96 96 96] Å grid dimension. We used a 10 Å cut-off radius for both Van der Waals and electrostatic interactions along with smooth particle mesh Ewald. [41] Our simulations were performed using NAMD-2.7 molecular dynamics software package [42] on 64 processors cluster.

## Supporting Information

Table S1 **Logistic regression analysis: status of individuals in function of associated haplotypes and their second order interaction.** DRB1-DQB1 haplotypes in MS patients and significant interaction factors (col.1), significance level (col.2), Odds Ratio (col. 3), 95% CI (col. 4).
(DOCX)
