# Supplementary material for: Interaction between HLA-DRB1-DQB1 Haplotypes in Sardinian Multiple Sclerosis Population
Source: PLoS One. 2013 Apr 8;8(4):e59790. doi: 10.1371/journal.pone.0059790 (PMC3620236; doi:10.1371/journal.pone.0059790)
Supplement: Table S1 — Logistic regression analysis: status of individuals in function of associated haplotypes and their second order interaction. DRB1-DQB1 haplotypes in MS patients and significant interaction factors (col.1), significance level (col.2), Odds Ratio (col. 3), 95% CI (col. 4). (DOCX) [file pone.0059790.s001.docx]

**Table S1.** Logistic regression analysis: status of individuals in function of associated haplotypes and their second order interaction. DRB1-DQB1 haplotypes in MS patients and significant interaction factors (col.1), significance level (col.2) , Odds Ratio (col. 3), 95% CI (col. 4).

| Haplotypes | p | OR | 95% CI |
| --- | --- | --- | --- |
| *03:01-*02:01 vs X | 1.74x10^-07^ | 1.7 | 1.4- 2.1 |
| *14:01-4-*05:031 vs X | 4.45x10^-01^ | 0.8 | 0.5-1.3 |
| *16:01-*05:02 vs X | 3.64x10^-03^ | 0.7 | 0.6-0.9 |
| *10:01-*05:01 vs X | 3.20x10^-03^ | 0.6 | 0.4-0.8 |
| *04:05-*03:01 vs X | 3.46x10^-07^ | 2.0 | 1.5-2.7 |
| *15:02-*06:01 vs X | 2.21x10^-06^ | 0.3 | 0.2-0.5 |
| *13:03-*03:01 vs X | 1.85x10^-05^ | 4.3 | 2.2-8.4 |
| *15:01-*06:02 vs X | 5.20x10^-03^ | 1.8 | 1.2-2.8 |
| *01 vs X | 5.14x10^-04^ | 0.7 | 0.5-0.8 |
| *07 vs X | 2.00x10^-01^ | 0.8 | 0.6-1.1 |
| *11 vs X | 7.10x10^-04^ | 0.7 | 0.6-0.9 |
| *12:01-*03:01 vs X | 2.30x10^-02^ | 0.6 | 0.4-0.9 |
| Interaction factor between *03:01-*02:01 and *14:01-*4-05:031 | 1.01x10^-03^ | 0.3 | 0.1-0.6 |
| Interaction factor between *03:01-*02:01 and *16:01-*05:02 | 9.22x10^-05^ | 0.5 | 0.4-0.7 |
| Interaction factor between *03:01-*02:01 and *07 | 4.89x10^-04^ | 0.4 | 0.2-0.6 |
| Interaction factor between *14:01-4-*05031 and *16:01-*05:02 | 2.14x10^-02^ | 0.3 | 0.1-0.8 |
| Interaction factor between *13:03-*03:01 and *11 | 1.20x10^-02^ | 0.2 | 0.1-0.7 |
| Interaction factor between *01 and *07 | 3.22x10^-02^ | 2.6 | 1.1-6.3 |
| Interaction factor between *01 and *011 | 3.73x10^-03^ | 2.1 | 1.3-3.4 |

Rare haplotypes belonging to the same haplogroup were grouped together: as *11 were designed *11:01-02-03-04 -*03:01. *11:01-*03:03-*05:02 and *11:04-*06:03; as *07 were designed *07:01- *02:01 and *07:01-*03:03; as *01 were designed *01:01 *05:01. *01:02-*05:01 and *01:03- *05:01.

X= all not associated haplotypes
